# Case report of a Japanese patient with chronic renal failure who developed SARS‐CoV‐2 in a hospital cluster during treatment for acute respiratory failure: An autopsy report

**DOI:** 10.1002/ccr3.6024

**Published:** 2022-07-11

**Authors:** Yoshifumi Amari, Satoshi Morimoto, Takashi Teranishi, Mai Ohata, Atsushi Takeshita, Hiroshi Hirano, Hitoshi Kobayashi

**Affiliations:** ^1^ Department of Nephrology Moriguchi Keijinkai Hospital Osaka Japan; ^2^ Department of Endocrinology and Hypertension Tokyo Women's Medical University Tokyo Japan; ^3^ Department of General Medicine Moriguchi Keijinkai Hospital Osaka Japan; ^4^ Department of Pathology and Laboratory Medicine Moriguchi Keijinkai Hospital Osaka Japan; ^5^ Department of Pathology and Laboratory Medicine Nozaki Tokushukai Hospital Osaka Japan

**Keywords:** autopsy, chronic renal failure, COVID‐19, diffuse alveolar damage, pathology, SARS‐CoV‐2

## Abstract

This article reports a clinical and histopathological perspective which noted not only COVID‐19 pneumonia but also exacerbation of chronic renal failure potentially caused by thrombus in the kidney, possibly COVID‐19‐related lesions. The accumulation of autopsy cases will elucidate the pathogenesis of COVID‐19 and aid in the development of effective therapeutics.

## Abstract 2

This article reports a clinical and histopathological perspective which noted not only COVID‐19 pneumonia but also exacerbation of chronic renal failure potentially caused by thrombus in the kidney, possibly COVID‐19‐related lesions.

## 1 INTRODUCTION

Coronavirus disease 2019 (COVID‐19), caused by the severe acute respiratory syndrome coronavirus 2 (SARS‐CoV‐2), has spread rapidly around the world since the end of 2019, responsible for 470 million infections and 6.1 million deaths as of March 2022.^1^ Despite the decrease in mortality rates and severity of COVID‐19 due to the development of vaccines and therapeutics, some individuals deteriorate rapidly and develop acute respiratory distress syndrome (ARDS).^2^ For immunocompromised patients, a history of smoking or underlying medical conditions (including chronic obstructive pulmonary disease [COPD], chronic kidney disease, diabetes, hypertension, cardiovascular disease, and obesity), COVID‐19 onset at any age is more likely to cause severe disease.^3^ A recent study found that the more risk factors which overlap for a patient, the more severe COVID‐19 is likely to be.^4^ Coronaviruses enter cells via the angiotensin‐converting enzyme 2 (ACE2), which is expressed in multiple tissues^5^ and disproportionately affects the respiratory system and other organs.^6^ Despite a huge number of reports that provide insights into COVID‐19, very little is known about the developmental mechanism of these pathological conditions. Salerno M et al. suggested that the lack of postmortem investigation did not allow a definition of the exact cause of death to determine the pathways of unknown infectious diseases.^7^ We report the clinical course and pathological autopsy findings for patients with COVID‐19 and chronic renal failure due to diabetic nephropathy and a history of prolonged smoking.

## 2 CASE PRESENTATION

**FIGURE 1:**
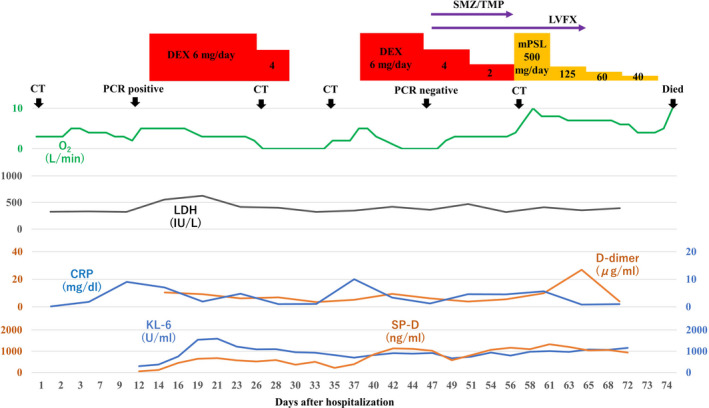
A summary of the clinical course with the essential laboratory findings

**FIGURE 2:**
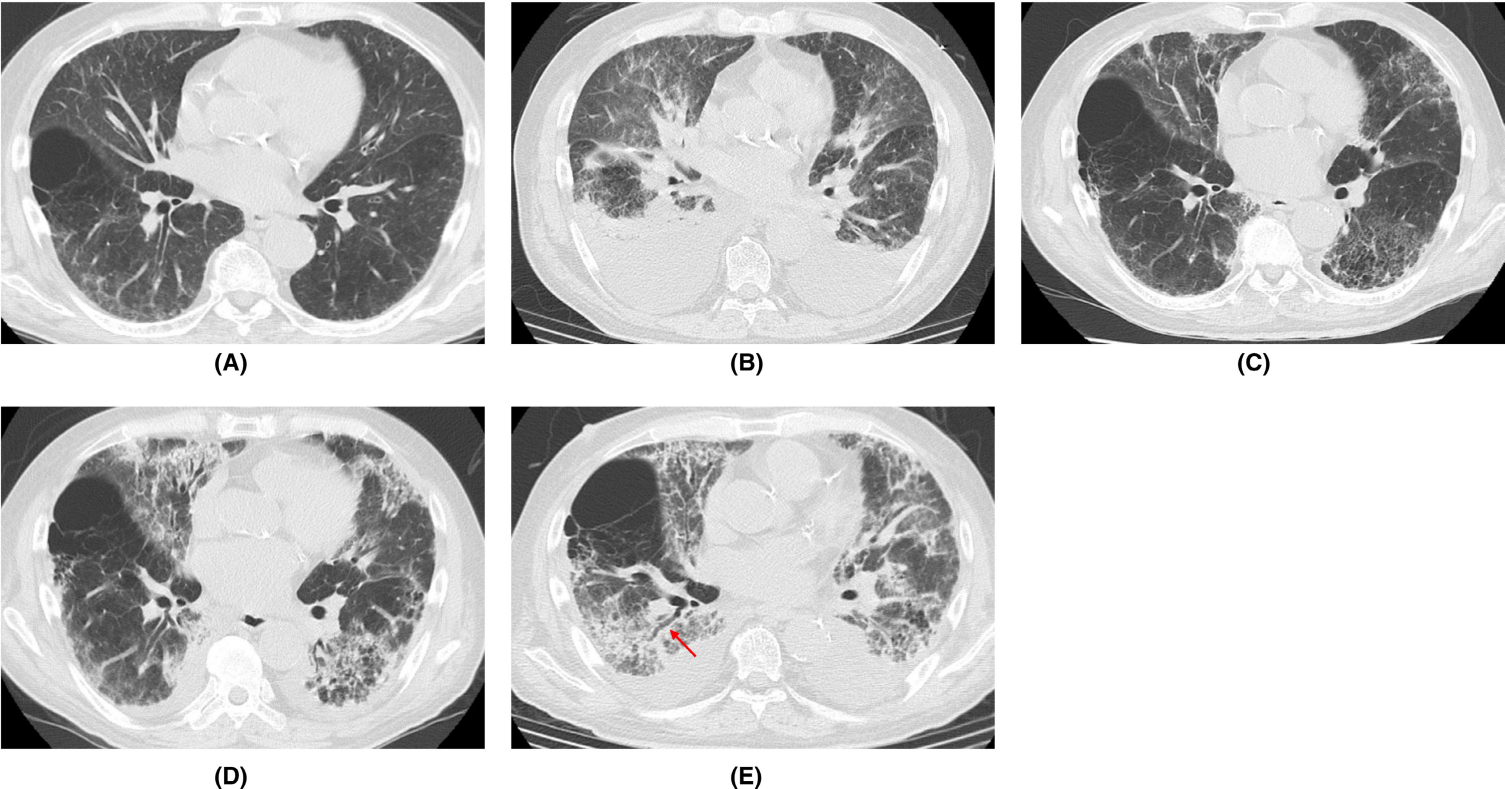
Representative images of thoracic CT. (A) Two months before admission; Showing no interstitial pneumonia image. (B) Day 1 (on admission); Showing bilateral pleural effusions, findings of pulmonary edema and interstitial edema. (C) Day 27 (19 days after positive PCR); Showing slight ground‐glass opacity. (D) Day 34 (26 days after positive PCR); Showing revealed bilateral ground‐glass opacities and infiltrative shadows. (E) Day 56 (48 days after positive PCR); Showing bilateral pleural effusions and aggravated lung shadows with traction bronchiectasis (arrow). CT, computed tomography; PCR, polymerase chain reaction

A 70‐year‐old man with hypertension, type 2 diabetes mellitus, and chronic renal failure due to diabetic nephropathy, arteriosclerosis obliterans in the lower extremities, and a history of prolonged smoking was transferred to the emergency department of our hospital with a chief complaint of shortness of breath. He was hospitalized for acute heart failure, likely due to chronic renal failure or valvular heart disease (moderate aortic valve stenosis). His respiratory condition improved after treatment with oxygen therapy and administration of diuretics. However, a cluster infection of SARS‐CoV‐2 occurred in our hospital. He developed a high fever on the eighth day after hospitalization (the same day as the cluster occurrence). The polymerase chain reaction (PCR) test (ID NOW, Abbott) using nasopharyngeal swab tests for SARS‐CoV‐2 on the ninth day was positive; therefore, we confirmed the COVID‐19 diagnosis. Thereafter, the patient was transferred to the isolation ward. On the tenth day, he required a high volume of oxygen and was diagnosed with severe COVID‐19. Along with COVID‐19 treatment (as outlined in the COVID‐19 Treatment Guidelines by National Institutes of Health),^8^ he was treated with steroid therapy (starting with 6 mg/day of dexamethasone) in combination with treatment for heart failure. Following an improvement of his respiratory symptoms, the patient worsened again after tapering off of steroid therapy, including exacerbation of renal failure (Creatinine values; 3.58–4.75 mg/dL). The patients did not respond to additional treatment (steroid pulse therapy and antibacterial agent). His status gradually worsened with declining oxygen saturation levels. He died of respiratory failure 74 days after hospitalization. A summary of the clinical course and essential laboratory findings is shown in Figure 1. Lactic acid dehydrogenase (LDH) values responded more acutely during the first exacerbation (increased oxygen requirement) (Day 9–16), and KL‐6 and hydrophilic surfactant protein D (SP‐D) values increased later. During the second exacerbation (Day 35–40), both LDH and KL‐6 values were stable at high levels, but the SP‐D value changed with the severity of the disease. The D‐dimer level was consistently high after the COVID‐19 infection. Thoracic computed tomography (CT) was performed once before admission and four times during hospitalization (Figure 2). No interstitial pneumonia was noted 2 months before admission. The major CT finding on admission was heart failure. After the COVID‐19 infection, the appearance of a slight ground‐glass opacity was observed, which gradually worsened.

**FIGURE 3:**
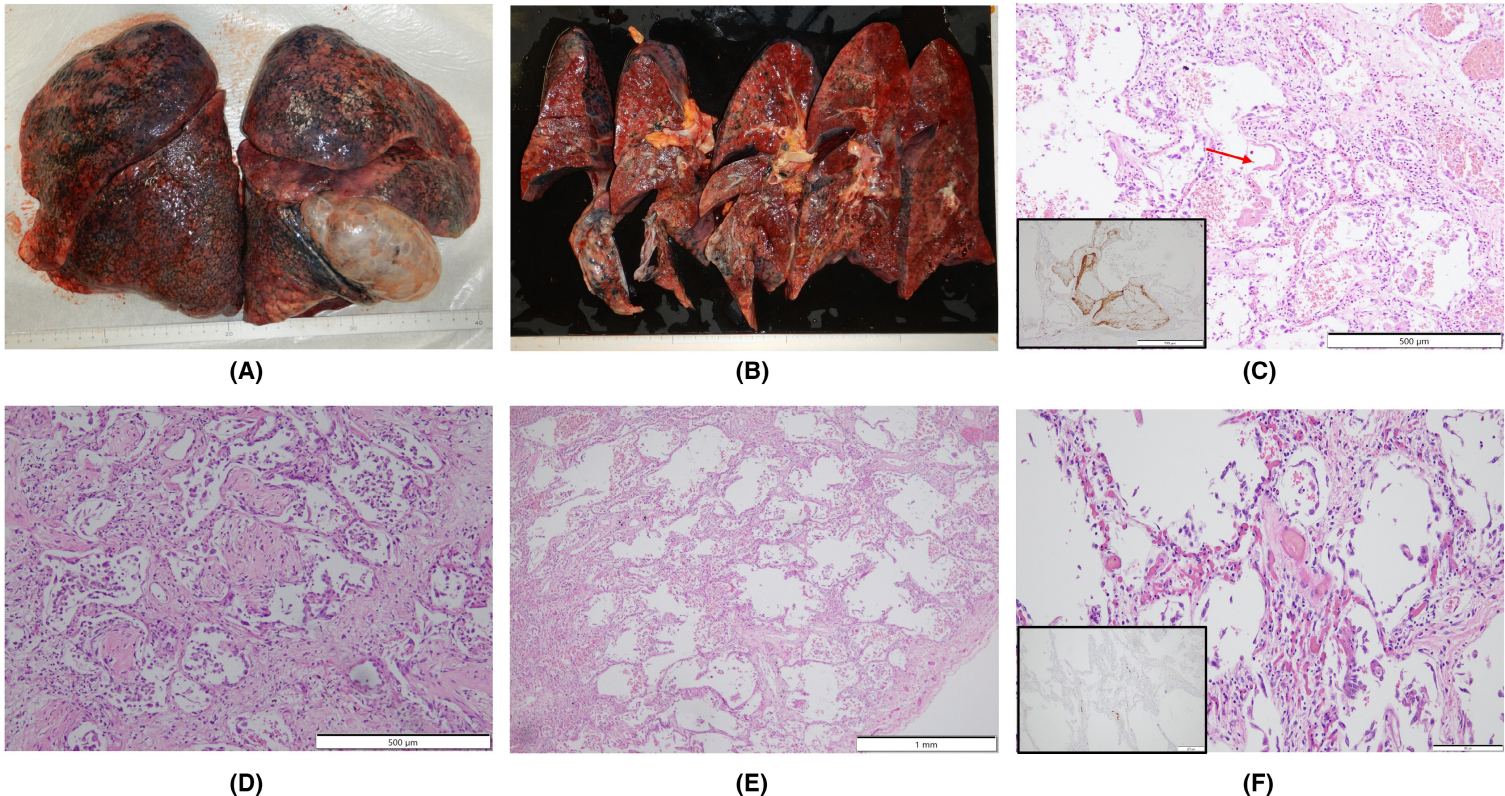
Macroscopic images and lung specimens of COVID‐19 pneumonia obtained from an autopsied case. (A) Both lungs are hyperinflated and appear to be cylindrical in shape, including overinflation of the upper lobe. (B) Gross appearance of transverse sections from the left lung. The cut surfaces also show the same cylindrical image as the whole image, due to overexpansion of the upper lobe. (C) Showing exudative phase of DAD with formation of hyaline membrane along alveolar septum (arrow), and intracapillary megakaryocytes are also present within hyaline membrane and were highlighted by CD 61 immunohistochemistry (inset). (D) Showing organizing phase of DAD with Masson body‐like structure including proliferation of fibroblast‐like cells and collagen fibers and fibroblast proliferation. (E) Showing fibrotic stage of DAD with alveolar wall thickening. (F) Showing intrapulmonary microthrombus highlighted by CD 61 immunohistochemistry (inset). CD 61, cluster of differentiation 61; COVID‐19, coronavirus disease 2019; DAD, diffuse alveolar damage

**FIGURE 4:**
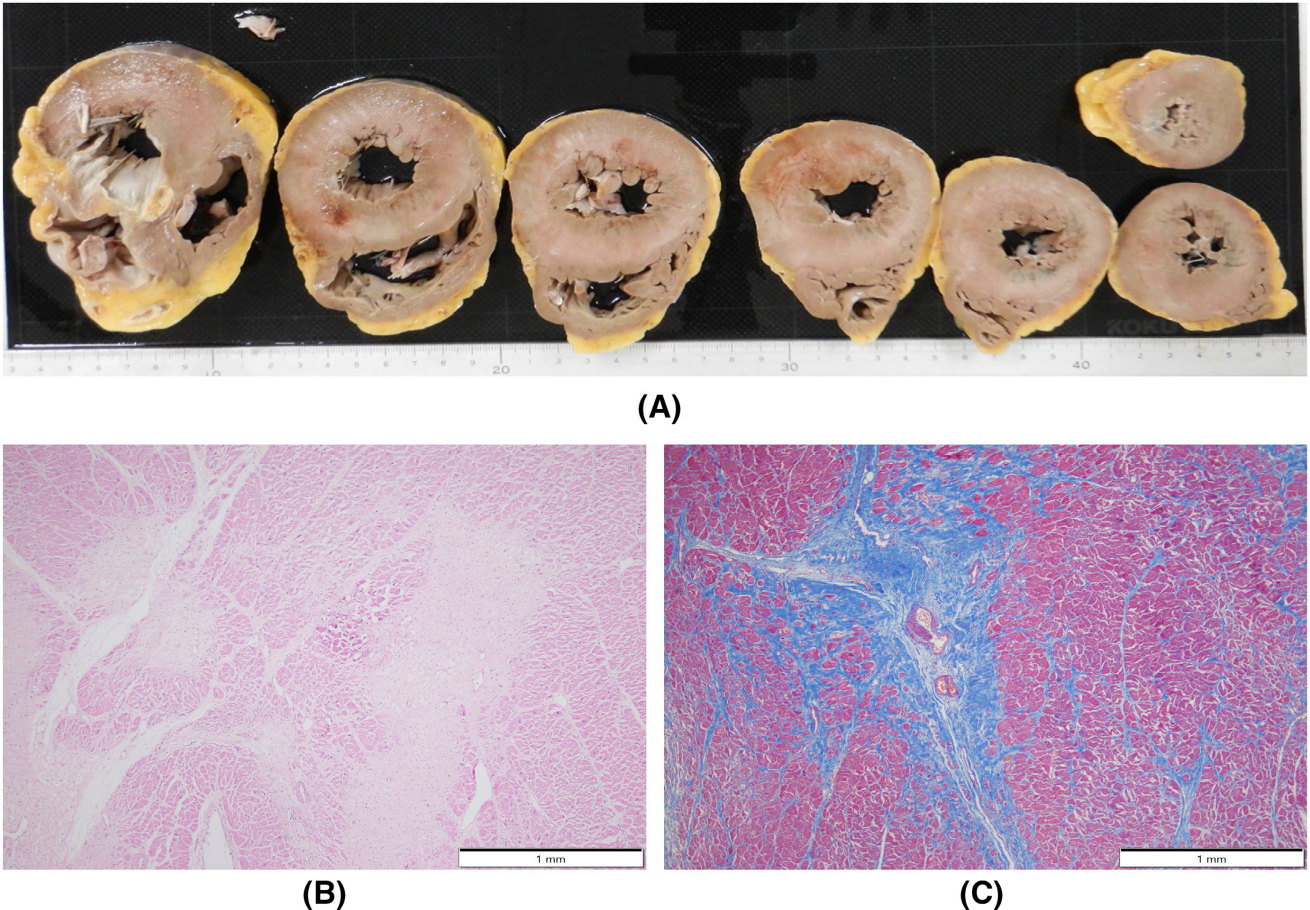
Macroscopic and histologic images of postmortem heart from patient who died with COVID‐19. (A) Afferent hypertrophy of the myocardium. (B, C) Cardiomyocyte shedding and fibrosis, highlighted by Masson trichrome immunohistochemistry. COVID‐19, coronavirus disease 2019

**FIGURE 5:**
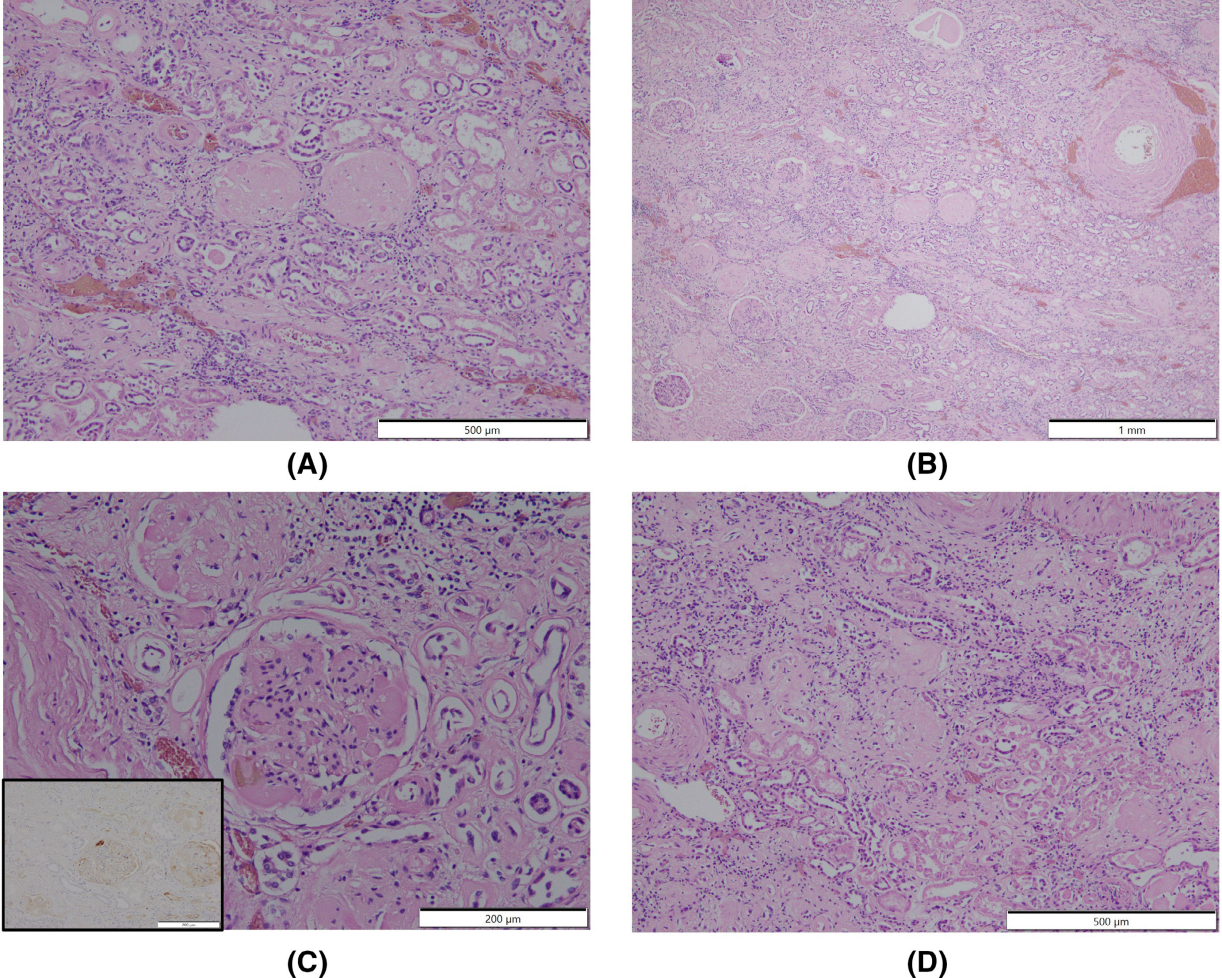
Histologic images of postmortem kidney from patient who died with COVID‐19. (A, B) Showing glomerular nodular lesions and diffuse glomerular lesions. (C) Microscopic thromboses in the glomerulus, highlighted by CD 61 immunohistochemistry (inset). (D) Showing acute renal tubular injury. COVID‐19, coronavirus disease 2019; CD 61, cluster of differentiation 61

An autopsy was performed 15 h postmortem. Table 1 summarizes the autopsy findings of our patient. The left and right lungs weighed 1040 and 1090 g, respectively. There was moderate charcoal dust deposition and a giant bra in the right middle lobe (Figure 3A). The upper lobes of both lungs were hyperinflated and appeared cylindrical in shape (Figure 3A). A gelatinous substance was found on the cut surface (Figure 3B). Hyaline membrane formation was mainly observed in the lower lobe of the right lung and the upper lobe of the left lung, which was positive for cluster of differentiation 61 (CD61), indicating diffuse deposition of fibrin‐like material (Figure 3C). Other findings included Masson's body‐like structures with fibroblast aggregation, thickened alveolar septum, and intrapulmonary microthrombus formation (Figure 3D–F). Macroscopically, the surface of the trachea exhibited erosions and histological examination revealed exfoliation and erosion of the epithelium of the trachea coated with fibrin. In addition, thromboses were observed in the kidneys, liver, and adrenal glands. The heart weighed 507 g. The cut surface exhibited concentric hypertrophy of the left ventricle macroscopically, and slight fibrosis was observed microscopically (Figure 4). There was no evidence of myocarditis or pericarditis, which have been reported to be associated with COVID‐19. Microscopic examination of the kidneys revealed nodular and diffuse lesions in the glomeruli, which were consistent with diabetic nephropathy (Figure 5A,B). Acute tubular damage was also observed in some tubules (Figure 5D).

**TABLE 1:** Summary of autopsy findings

Pathological anatomical diagnosis	
Major lesion	1) COVID‐19 pneumonia (DAD)
	2) COVID‐19‐related lesions (intrarenal thrombosis, intrahepatic thrombosis, and intraparanephric thrombosis)
Minor lesion	1) Left ventricular hypertrophy, coronary arteriosclerosis (left anterior inferior branch), and myocardial focal fibrosis (moderate)
	2) Cardiac cyst fluid storage (yellow transparent, 450 mL)
	3) Liver congestion
	4) Atherosclerosis of aorta (high)
	5) Diabetic nephropathy, intrarenal arteriosclerosis (mild)

Organs	Weight	Pathological findings	Immunohistological techniques; CD61‐positive material (fibrin)
Lung	1040 g (left), 1090 g (right)	Pulmonary capillary congestion, DAD (exudative), DAD (proliferative), microthrombi of alveolar capillaries	(+)
Kidney	120 g (left), 100 g (right)	Diabetic nephropathy, microthrombi in the glomerulus	(+)
Heart	507 g	Myocardial hypertrophy	(−)
Liver	1360 g	Liver congestion	(+)
Pancreas	70 g	Pancreatic atrophy	(−)
Adrenal glands	5 g	No remarkable change	(+)
Spleen	134 g	Splenomegaly	(−)

Abbreviations: CD61, cluster of differentiation 61; COVID‐19, coronavirus disease 2019; DAD, diffuse alveolar damage.

## 3 DISCUSSION

The pathophysiology of death with respect to COVID‐19 is not clearly defined despite the increasing number of published papers on COVID‐19. The most common comorbidities in COVID‐19 patients reported in previous reports were cardiovascular disease, metabolic abnormalities (diabetes and obesity), respiratory disease (chronic obstructive pulmonary disease), and cancer; the most frequently reported complications were ARDS, acute kidney injury, myocardial infarction, liver failure, and septic shock.^7^ Our patient also had hypertension, arteriosclerosis obliterans in the lower extremities, and type 2 diabetes mellitus as comorbidities, and ARDS and acute exacerbation of chronic renal failure as complications. With regard to the acute exacerbation of chronic renal failure, it was suspected that microemboli in the renal tissue may cause the disorder. The patient was a smoker, was over 65 years old, and had chronic kidney disease and hypertension, and had an unfortunate outcome in accordance with a previous report indicating that with overlapping risk factors, COVID‐19 is likely to be more severe.^4^ High D‐dimer levels, considered to be a marker of the severity of COVID‐19,^9^ were present after SARS‐CoV‐2 infection (Figure 1). KL‐6 and SP‐D are frequently used as serum markers for interstitial pneumonia, and both are produced by alveolar type II epithelial cells in the lungs.^10^ As for the time of elevation, SP‐D is considered to be elevated earlier and is useful for follow‐up in the acute phase or at the time of recurrence.^11^ In this case, SP‐D level was decreased by steroid administration, but increased upon tapering the treatment. Although the elevation of LDH is considered to be a nonspecific indicator of lung injury, in this case, the fact that LDH was elevated earlier than KL‐6 and SP‐D was considered to be a unique feature. Chest CT findings of COVID‐19 pneumonia is characterized by the fact that the lesions are more often seen as bilateral shadows, distributed on the periphery of the lung field, and predominantly in the lower lobes.^12^ Aggravation of COVID‐19 pneumonia leads to the pathogenesis of ARDS.^13^ In the acute exudative stage of ARDS, consolidation and ground‐glass shadow attenuation are seen on CT, and in the subacute proliferative stage, traction bronchiectasis, lung shrinkage, and distortion are seen inside the consolidations; remodeling of lung structure progresses in the chronic fibrosis stage, resulting in the development of destroyed honeycomb lung. ARDS is characterized by a mixture of these phases in various proportions at the time of diagnosis after injury, and the findings of consolidation and ground‐glass shadow attenuation with traction bronchiectasis are particularly important because they indicate a combination of acute exudative and subacute proliferative phases. In this case, the findings of the last chest CT were consistent with these features (Figure 1E).

Diffuse alveolar damage (DAD) is a histological pattern of ARDS.^8^ DAD develops simultaneously and progresses to the exudative, proliferative (organizing), and fibrotic phases over time.^14^ One major feature of COVID‐19 pneumonia is the simultaneous presence of these lesions at different stages within the same individual lung lobe.^15^ In this case, pulmonary hyaline membrane formation, which was revealed in the acute (exudative) stage of DAD, was observed in part of the alveolar lumen (Figure 3C). Pulmonary hyaline membrane formation often appears 3–7 days after lung tissue damage,^14^ but in this case, even though the acute phase had already passed, the acute phase findings of DAD were also confirmed. Masson's body (a collection of fibroblasts) is a common finding in the subacute proliferative and organizing phases of DAD, and pulmonary fibroblast growth is also a common finding in the subacute stage of DAD.^16^

These findings were seen in this case, which seems to be due to the comparatively long clinical course. A thickened alveolar septum is a common finding in interstitial pneumonia, but it also appears in the fibrosis stage of DAD.^17^ In this case, fibrosis inside and outside the alveoli may have been observed due to the elapsed time of more than 2 weeks after COVID‐19 onset. DAD is thought to be expressed in all lung lobes but is more prevalent in the middle and lower lung fields.^18^ In this case, the upper lobes of both lungs were hyperinflated (Figure 3A,B), which was presumably caused by the flow of aspirated air into the upper lobe as the lesion developed mainly from the lower lobe and progressed to fibrosis. The pathological findings in this case are summarized as follows: The main lesions in both lungs were in the lower lobes, various stages of DAD were observed histologically, and airway lesions were confirmed, all of which are consistent with the representative findings of COVID‐19 pneumonia. COVID‐19 has been reported to cause a variety of thrombotic complications.^19^ In this case, thromboses were confirmed in multiple organs, including the kidneys, liver, and adrenal glands, as well as the lungs, suggesting that these conditions had an adverse effect on the patient's prognosis. Excessive inflammation and vascular endothelial cell damage have been reported as causes of COVID‐19‐related thrombosis.^20^ It has been pointed out that viral infection of endothelial cells may cause endothelial injury because ACE2, the receptor of SARS‐CoV‐2, is expressed in the vascular endothelium.^21^ As described by Cipolloni L et al, histological and immunohistochemical investigations revealed the presence of the virus within endothelial cells, promoting an inflammatory response.^22^ It has also been reported that the levels of factor VIII and VWF are elevated in the blood of patients with COVID‐19.^23^ Factor VIII activated by thrombin composes tenase, an activating complex that plays a role in protein coagulation and induces coagulation in activated platelets. However, the definitive pathogenesis is still unknown, and it should be considered a specific coagulation abnormality. Further studies are required to investigate this in greater detail. Menter T et al. reported the autopsy findings of extrapulmonary organs involved in COVID‐19, including myocardial hypertrophy (71.4%), senile amyloidosis (28.6%), acute myocardial cell necrosis (14.3%), acute myocardial infarction (4.8%), acute tubular damage (93.3%), disseminated intravascular coagulation (17.6%), hypertensive nephropathy (11.8%), and diabetic nephropathy (11.8%) in the kidney.^24^ In this case, we also found myocardial hypertrophy, acute tubular damage, and diabetic nephropathy, but all of these findings are nonspecific and difficult to link to COVID‐19. Regarding the heart, myocarditis, pericarditis, and microthrombi have been reported as typical pathological findings after COVID‐19 infection,^25^ which were not observed in this case. Regarding the kidneys, macroscopically, the gross appearance of transverse sections was consistent with the image of nephrosclerosis associated with hypertension, and histologically, glomerular nodular lesions and diffuse glomerular lesions were consistent with the image of diabetic nephropathy. Thrombus deposition was observed in the glomeruli and renal capillaries, which may be involved in the worsening of chronic renal failure during the course of hospitalization. In a previous report, proximal tubular damage was reported as a characteristic of kidney pathology in autopsies of COVID‐19 cases.^26^ Proximal tubular damage was also observed in this case, but it could not be determined whether it was due to COVID‐19 infection or other causes.

## 4 CONCLUSION

In conclusion, we presented the autopsy of a patient with chronic renal failure who developed SARS‐CoV‐2 in a hospital cluster during treatment for acute respiratory failure. The accumulation of autopsy cases is expected to elucidate not only the pathogenesis specific to COVID‐19 but also the common pathogenesis of ARDS associated with viral pneumonia, and aid in the development of effective therapeutics.

## AUTHOR CONTRIBUTIONS

Yoshifumi Amari: wrote the manuscript, collected the data, Takashi Teranishi, Mai Ohata, Atsushi Takeshita, Hiroshi Hirano, and Hitoshi Kobayashi: collected the data, and Satoshi Morimoto: drafted and revised the paper.

## CONFLICT OF INTEREST

The author(s) declare no potential conflict of interest with respect to the research, authorship, and/or publication of this paper.

## ETHICAL APPROVAL

The study was published with written consent of the patients.

## CONSENT

Written informed consent was obtained from the patient's next of kin to publish this report in accordance with the journal's patient consent policy.

## Data Availability

Data sharing not applicable to this article as no datasets were generated or analysed during the current study.
